# α-Glucosidase inhibition by green, white and oolong teas: *in vitro* activity and computational studies

**DOI:** 10.1080/14756366.2023.2236802

**Published:** 2023-07-20

**Authors:** Fabio Esposito, Nicolino Pala, Mauro Carcelli, Samuel T. Boateng, Paolo S. D’Aquila, Alberto Mariani, Sandro Satta, Jean Christopher Chamcheu, Mario Sechi, Vanna Sanna

**Affiliations:** a Department of Medicine, Surgery and Pharmacy, Laboratory of Drug Design and Nanomedicine, University of Sassari, Sassari, Italy; b Department of Chemistry, Life Sciences and Environmental Sustainability, University of Parma, Parma, Italy; c School of Basic Pharmaceutical and Toxicological Sciences, College of Pharmacy, University of Louisiana at Monroe, Monroe, LA, USA; d Department of Biomedical Sciences, University of Sassari, Sassari, Italy; e Department of Chemical, Physical, Mathematical and Natural Sciences, University of Sassari, Sassari, Italy; f Department of Medicine, David Geffen School of Medicine, University of California, Los Angeles, CA, USA; g Nanomater S.r.l, Alghero, Italy

**Keywords:** α-glucosidase inhibition, tea, polyphenols, catechins, molecular docking

## Abstract

Natural α-glucosidase inhibitors from plant-based foods such as catechins offer an attractive strategy for their potential anti-diabetic effects. In this study, infusions of three different tea types (green, white, and oolong) were investigated for their total phenolic (TPC) and catechins (EGCG, ECG, EGC, and EC) content, and for their α-glucosidase inhibitory activities. We observed that the level of TPC in white tea was significantly higher compared to oolong and green tea, which suggests higher content of EGCG and ECG catechins in fresh young leaves. Our findings showed that the higher content of such catechins in the infusion of white tea well correlated with a strong inhibition of α-glucosidase, and such inhibition was demonstrated to be more effective than the FDA-approved drug acarbose. Then, we computationally explored the molecular requirements for enzyme inhibition, especially for the most active catechins EGCG and ECG, as well as their disposition/stability within the active site.

## Graphical Abstract

**Figure:**
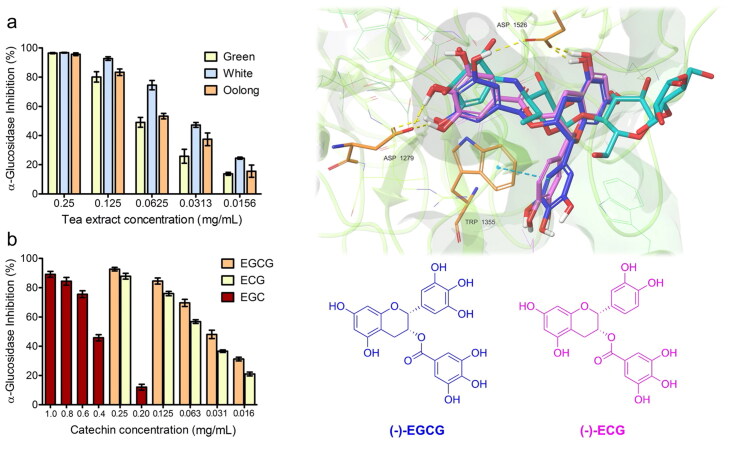

## 1. Introduction

The prevention of the fast breakdown of sugars, and the control of postprandial hyperglycaemia could have potential anti-diabetic effects and represents an efficient therapeutic approach to manage diabetes mellitus, especially type 2^1^^,^^2^. α-glucosidase inhibitors moderate plasma triglycerides levels, cardiovascular disorders, and hypertension risks, reducing glucose and improving body insulin response^3–5^. Currently, the prescribed α-glucosidase inhibitor drugs, acarbose, miglitol or voglibose, are often associated with side effects such as diarrhoea, flatulence, and abdominal pain, which limit their long-term administration^6^^,^^7^. In this context, plant-based foods or dietary supplements have received particular interest as an alternative approach to α-glucosidase inhibitors due to their low cost, and relative safety, including a low incidence of gastrointestinal side effects^6–9^.

Previous studies indicated that different tea extracts and catechins have inhibitory activity against two of the main carbohydrate digestive enzymes, α-amylase and α-glucosidase, which are involved in starch breakdown and intestinal glucose absorption^10^^,^^11^. In particular, some evidence suggest that tea extracts and catechin 3-gallates, especially *(-)-epigallocatechin-3-gallate* (EGCG), were less effective inhibitors of α-amylase, but demonstrated potent inhibition of α-glucosidase^12–14^.

Because of the beneficial properties of tea polyphenols in human diseases such as diabetes, cardiovascular disease, and neurological disorders, as well as in cancer prevention, a diverse spectrum of different cell types and mechanisms is involved in the molecular effects of these compounds^15^. The mechanisms underlying the molecular action of catechins involved in inducing or suppressing the activity of various transcription factors, interfering at different levels of growth factors regulation, regulating signalling pathways, scavenging free radicals by their anti-oxidative properties and activation of the detoxification system, and further contributing to changes in enzyme activities, such as protein kinases^15–17^. As far as the cellular and molecular mechanisms of EGCG are concerned, its *in vitro* and vivo pharmacological profiles suggested that its chemopreventive/antiproliferative action is mediated by interaction with specific biological targets involved in the regulation of crucial steps of cell proliferation, carcinogenic and metastatic processes. However, several proteins have been identified as putative EGCG direct targets. For example, the trans-membrane 67 kDa laminin receptor (67LR), a master regulator of many pathways affecting cell proliferation or apoptosis, has been identified as a high-affinity EGCG receptor^18^. EGCG was also found to be interacting directly with the peptidylprolyl cis-/trans isomerase A1 (Pin1), the transforming growth factor-beta (TGF-β) receptor type 2 (TGFR-II), and some metalloproteinases (MMPs). Moreover, EGCG was found to interact with protein phosphatase-2a and modulates p53-bak apoptotic pathway^19^, and also would interact with DNA methyltransferases (DNMTs) and histone deacetylases (HDACs). Collectively, this information about the mechanisms of action of EGCG, together with novel insights can contribute to explaining its onco-suppressive function^17^^,^^20^.

Concerning the anti-diabetic effects, the potential for green and white teas to promote glucose and lipid metabolism was further confirmed in HepG2 cell lines^21^ and *in vivo* studies^22^^,^^23^. Kong et al.^24^ demonstrated that the antioxidant activity and inhibitory potential of α-amylase and α-glucosidase of teas were related to their degree of processing and the level of oxidation, with a better activity for teas that had none or slight fermentation.

White, green, oolong and black teas are all derived from the Camellia sinensis plant, and they are the major varieties consumed. Differences among the four types of tea result from the various degrees of processing and the level of oxidation, influencing the concentration of catechins^25^. Unfermented green tea is produced from the young leaves that are plucked, withered, rolled, and then immediately heated to halt oxidation. White tea, less oxidised than green tea, is made from the youngest leaves or new growth buds that are steamed and gently air dried immediately after picking to prevent the oxidation process^26^. The semi-oxidised oolong tea is produced with a shorter fermentation period and oxidation may range from 12–85%. Black tea is further processed and fully fermented, the leaves are initially dried and rolled and then exposed to heat, light and crushed^27^.

Further studies on digestive enzyme inhibition were carried out on different tea extracts produced in conditions that are not comparable to the conventional preparation methods used by consumers^28^^,^^29^. It is known that the solvent extraction and used conditions considerably affect the quantity and quality of bioactive components and consequently the potential hypoglycaemic effect of tea. Again, Miao et al.^30^ investigated interactions of catechins with pancreatic α-amylase and the structural requirements for inhibitory activity. However, the correlation between the structure and the activity of catechins as natural inhibitors of α-glucosidase has not yet been clarified.

In this context, we compared the total phenolic and catechins content, and α-glucosidase inhibition activity of unfermented green and white tea, and half-fermented oolong tea infusions (GTI, WTI, and OTI, respectively). Moreover, to correlate the α-glucosidase inhibition of tea extracts with their catechins content, the enzyme inhibition activity for individual catechins was determined. Furthermore, we investigated the interactions between single catechins and α-glucosidase using computational docking to understand the molecular requirements for enzyme inhibition.

## 2. Results and discussion

### 2.1. Preparation and characterisation of tea extracts

Infusion of three different tea types, (GTI, WTI, OTI), was carried out using water as a solvent, a ratio of water-to-tea of 20:1 ml/g, at 100 °C, and 5 min extraction time. As reported in Table 1, the yields of extract for white tea (about 350 mg/g dry weight of tea leaves) were significantly higher (*p* < 0.05) compared to green and oolong teas (268 and 284 mg/g dry tea leaves, respectively). We previously observed that the white tea infusion (1.0 g of leaves in 20 ml of distilled water) at 60 °C for 30 min produced an average of 291.33 ± 6.11 mg/g dry weight of tea leaves, suggesting that the increase of temperature and the reduction of time has a favourable impact on the efficiency of the extraction process^31^. In terms of human consumption, tea represents a major source of dietary polyphenols (30 to 40% wt/wt of extract solids), mainly responsible for colour and astringency^32^.

**Table 1.** Yields of extract (expressed as mg/g dry weight of tea leaves), TPC (expressed as mg GAE/g extract), and percentage of TPC/g dry weight of tea leaves.

Samples	mg extract/g tea	TPC (mg GAE /g extract)	TPC/g leaves (%)
GTI	268 ± 6.0	74.36 ± 1.54*	27.75 ± 0.62
WTI	346 ± 12.0*	93.98 ± 0.56*	27.16 ± 0.94
OTI	284 ± 12.2	89.15 ± 0.64*	31.39 ± 1.35*

* Significantly different from each other tea.

All prepared tea extracts were compared for their TPC, in terms of GAE. As reported in Table 1, the level of TPC was found to be significantly higher in the white tea (93.98 ± 0.56 GAE mg/g), followed by oolong (89.15 ± 0.64 GAE mg/g), and then green tea (74.36 ± 1.54 GAE mg/g), corresponding to a percentage of TPC/g dry weight of tea leaves of about 28% for green and white, and 31% for oolong tea.

### 2.2. Content of major catechins ECG, EC, EGCG, and EGC in green, white, and oolong tea infusions

**Figure 1.**
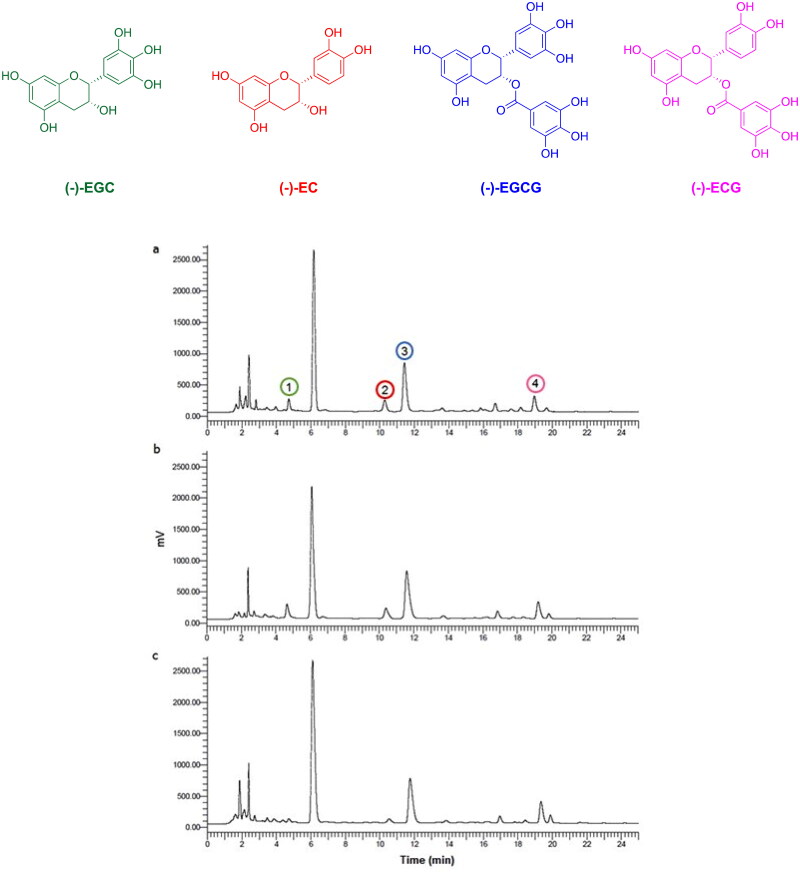
Chemical structure of catechins and HPLC separation of green (a), white (b) and oolong (c) tea extract (1: EGC, 2: EC, 3: EGCG, 4: ECG).

The individual catechins in different tea extracts were identified and quantified by HPLC and a representative chromatogram is presented in Figure 1. The retention time of *(-)-epigallocatechin gallate* (EGCG), *(-)-epigallocatechin* (EGC), *(-)-epicatechin gallate* (ECG), and *epicatechin* (EC) resulted in 4.3, 10.5, 11.8, and 19.3 min, respectively.

The contents of catechins, the dominant phenolics in tea leaves, confirmed that white, green, and oolong teas are rich sources of bioactive compounds (see Table 2). The total concentration of the investigated catechins was similar in WTI and OTI (∼47 mg/g tea leaves), and the results were significantly higher compared to the amount detected in GTI (∼38 mg/g tea leaves). Normally, green tea has been reported to contain a larger amount of catechins than oolong tea.

**Table 2.** Content of major catechins ECG, EC, EGCG, and EGC (expressed as mg/g dry weight of tea leaves) in green, (GTI) white (WTI), and oolong (OTI) tea infusions.

Catechin (mg/g)	GTI	WTI	OTI
ECG	2.1 ± 0.9	7.5 ± 0.8*	3.2 ± 0.7
EC	2.8 ± 0.1	1.9 ± 0.6	4.7 ± 0.6*
EGCG	21.7 ± 1.3	32.3 ± 1.4*	21.1 ± 0.3
EGC	11.3 ± 0.9*	5.2 ± 0.5*	17.9 ± 1.3*
Total	37.8 ± 1.4*	46.7 ± 3.3	46.8 ± 0.9

*Significantly different from each other tea

These unexpected results could be related to the influence of factors such as season, location, climate, cultivar type, maturity of the leaf, manufacturing practices and process on the catechin content in different tea leaves^33^^,^^34^. Additionally, storage for an extended time can lead to loss of quality of tea because catechins are not stable during long-term storage^35^.

According to the literature, the dominant catechin, EGCG, ranged from 21 mg/g dry weight in green and oolong tea leaves compared to 32 mg/g in white tea^36^. Moreover, the white tea, primarily manufactured from the young apical hairy bud, showed a higher content of EGCG and ECG, and in larger amounts in fresh young leaves.

### 2.3. α-Glucosidase inhibition activity

The inhibition of α-amylase and α-glucosidase enzymes responsible for carbohydrate digestion can be an important approach in the management of blood glucose levels in type 2 diabetic and borderline patients^37^. Previous studies suggested that tea extracts and catechins were considerably less potent than commercial acarbose in inhibiting α-amylase activity, however, they were much more potent inhibitors of α-glucosidase^12^.

**Figure 2.**
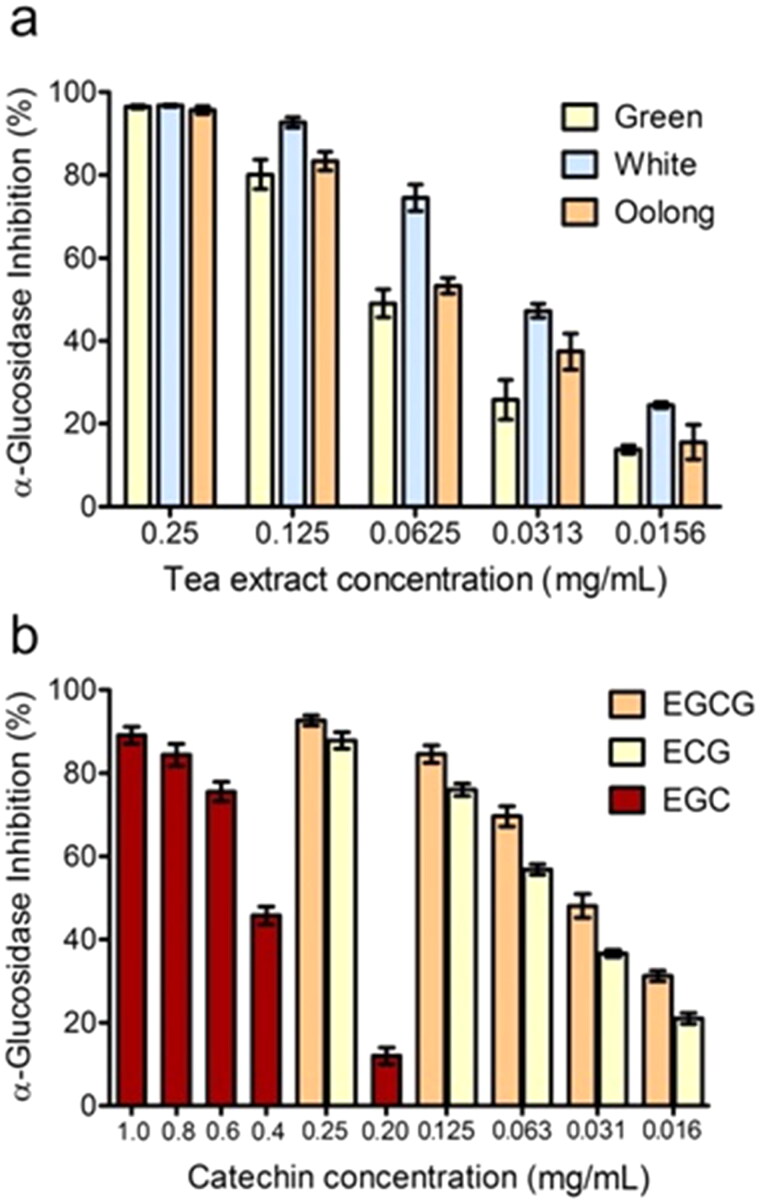
α-glucosidase inhibitory effect of the tea extracts (a), and standard catechins (EGCG, ECG, and EGC) (b) at different concentrations.

In the present study, we focused on the α-glucosidase inhibition activity of different tea extracts and isolated catechins, in comparison with acarbose. As depicted in Figure 2(a), the α-glucosidase inhibition capacity of tea extracts is concentration-dependent.

At the concentration of 0.25 mg/mL, a complete inhibition of the α-glucosidase activity was observed for all three tea extracts. In the range of 0.125 to 0.0156 mg/mL concentration, the inhibition capacity decreased in the following order: white > oolong > green tea extract, with IC_50_ values of 42.0 ± 1.8 μg/mL, 63.1 ± 4.2 μg/mL, and 71.6 ± 3.8 μg/mL, respectively (Table 3).

**Table 3.** α-Glucosidase Inhibition activity by the tea infusions (GTI, WTI, OTI), standard catechins (EGCG,ECG, EGC, EC), and commercial acarbose expressed as IC_50_ values.

Sample	IC_50_ (mg/mL)
GTI	0.072 ± 0.004
WTI	0.042 ± 0.002
OTI	0.063 ± 0.004
EGCG	0.042 ± 0.002
ECG	0.053 ± 0.003
EGC	0.418 ± 0.001
EC	Nd
Acarbose	1.25 ± 0.02

Nd: Not determined

Interestingly, the calculated IC_50_ values of WTI, GTI, and OTI for α-glucosidase activity were 17, 30, and 20 times higher than those of acarbose (1.25 mg/mL)^38^. These findings suggest that all tested tea extracts strongly suppress the α-glucosidase and can be beneficial for the control of postprandial hyperglycaemia.

In order to correlate the α-glucosidase inhibition of tea extracts with their catechins content, the test was also performed on individual catechins. As shown in Figure 2(b), some of the catechins tested exerted potent inhibitory effects on α-glucosidase activity. For EGCG and ECG the inhibition activity was observed in the concentration range of 0.25–0.016 mg/mL. In contrast, for the nongallated catechins, EGC showed an α-glucosidase inhibitory capacity at higher concentrations (1.0–0.20 mg/mL), while the activity of EC was not detected^12^. The calculated IC_50_ values of EGCG, ECG, and EGC for α-glucosidase activity resulted 30, 26, and 3 times higher than those of acarbose (Table 3).

In agreement with the literature^39^, our results suggest that the α-glucosidase inhibitory activity of different tea extracts was correlated with total phenolic and specific catechin contents. Particularly, in this study, the higher inhibition of WTI could be due to its higher total phenolic content (93.98 ± 0.56 mg GAE/g extract) as well as the increased levels of EGCG and ECG, contributing to the inhibitory effect on α-glucosidase.

### 2.4. Computational studies

In order to characterise, at the molecular level, the putative inhibitory interaction between the tea catechins EGCG, EGC, ECG, and EC and the target enzyme α-glucosidase, molecular docking studies were performed. Although α-glucosidase possesses two catalytic centres, located at the N-terminal (indicated as αG-N) and C-terminal (αG-C) subunits, respectively, we focused our study on the αG-C as the depositary of the highest enzymatic activity^9^. Structurally, αG-C is a ∼100 kDa weighted subunit composed of five distinct domains where the catalytic one is formed by the central residues 1221–1632, which accounts for approximately 50% of the total volume.

Using Autodock Vina v1.2.3 (AV)^40^ as a first method for docking calculations, we first validated the calculation by re-docking co-crystallized acarbose as the reference drug. The binding energy and root mean square deviation (RMSD) results obtained (–8.54 kcal/mol and 1.52 Å, respectively) corroborated the docking software capability to predict the binding conformation of the acarbose co-crystal structure. Tea catechins free binding energies were −9.47, −9.44, −8.32, −8.40 kcal/mol, for EGCG, ECG, EGC and EC, respectively.

To further assess the interactions between tea catechins and α-glucosidase, molecular docking was performed using AutoDock v4.2.6 (AD4)^41^. After AD4 re-docking of acarbose (binding energy was −4.75 kcal/mol, with a RMSD of 1.26 Å), calculations for tea catechins provided a trend comparable with that obtained with AV in terms of free binding energy results. Specifically, we obtained −6.46, −6.14, −5.45, −4.82 kcal/mol values for EGCG, ECG, EGC and EC, respectively.

**Figure 3.**
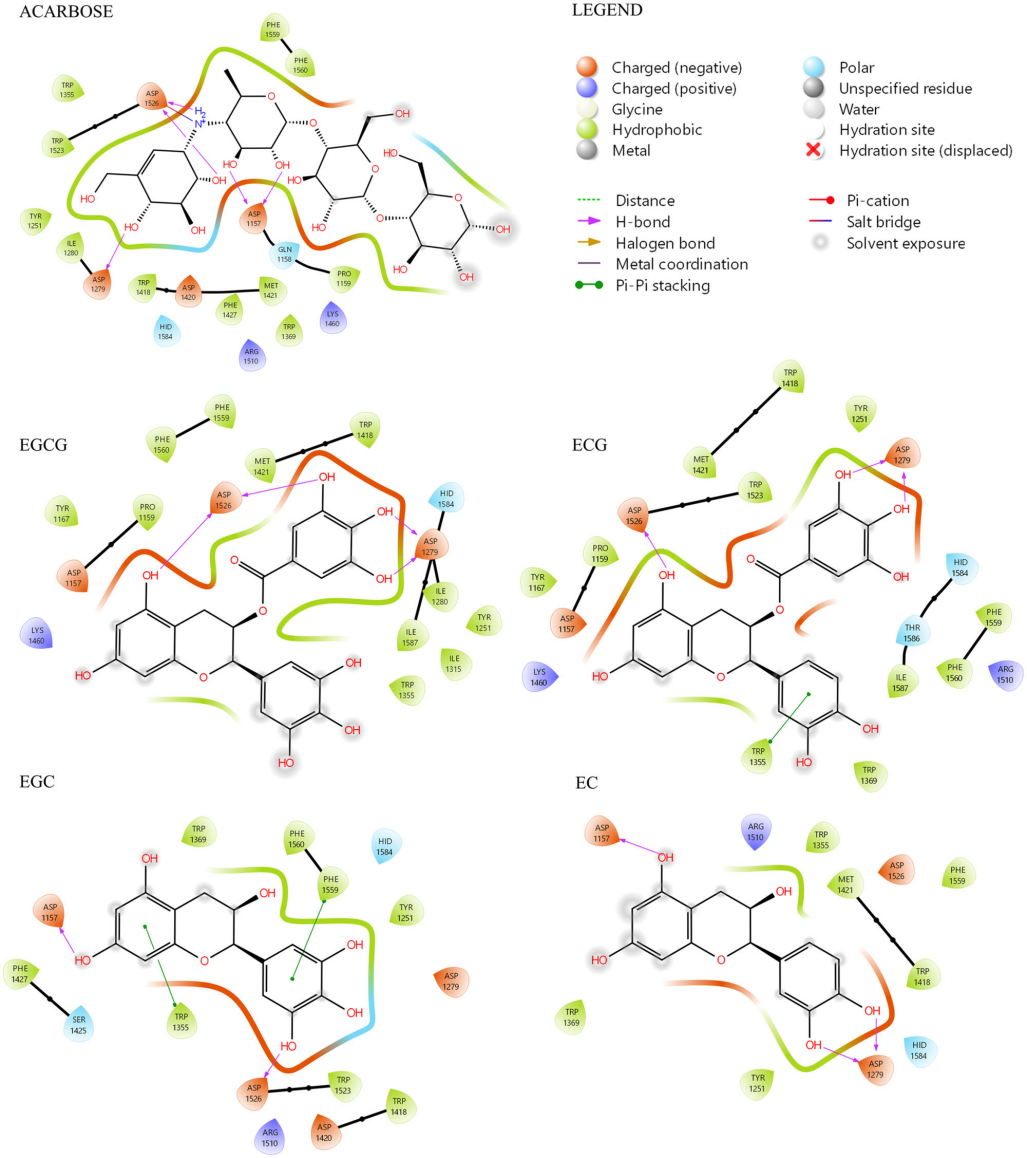
Ligand interaction maps of co-crystallized acarbose and the docked compounds EGCG, EGC, EGC, and EC, obtained from AD4 calculation.

Ligand interaction maps of EGCG, ECG, EGC, EC, and acarbose highlighted the pattern of amino acid residues that are involved in the ligand-protein complexes. As shown in Figure 3, acarbose can establish peculiar interactions with the enzyme. Only three of the four rings of acarbose were positioned within the catalytic site, where the cyclohexene ring located on the bottom of the pocket is involved in three H-bonds with Asp 1279, Asp 1526, and one salt bridge with Asp 1526. Two additional H-bonds are established by the adjacent ring with Asp 1157. The fourth ring is totally exposed to the solvent and does not seem to have significant interactions with the enzyme. Concerning the Ligand Interaction maps obtained for each catechin, the two catechins with the highest binding energies, EGCG and ECG (-6.46 and −6.14 kcal/mol, respectively), established consistent interactions with the enzyme.

**Figure 4.**
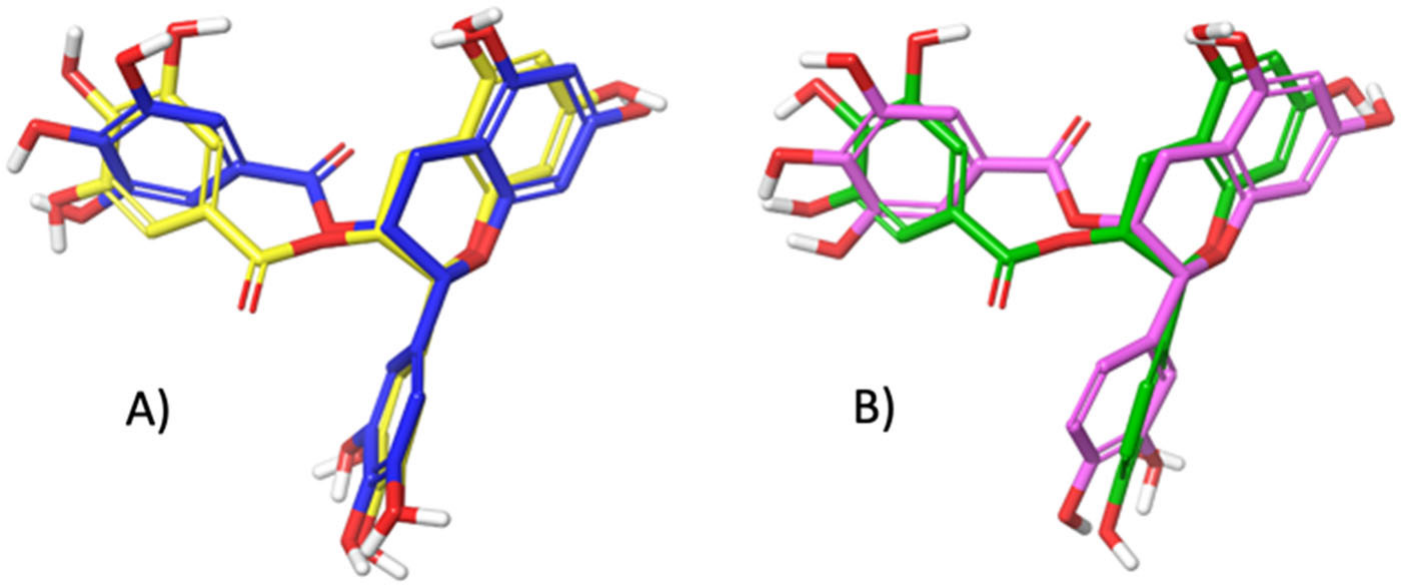
A) Superimposition between the best pose of EGCG obtained with AV (in yellow) and AD4 (in blue). B) Superimposition between the best pose of ECG by AV (in green) and AD4 (in violet).

As shown in Figure 4, both software provided for EGCG and ECG a very similar tridimensional conformation for the best pose (RMSD for EGCG: 1.20 Å, Figure 4(A); RMSD for ECG: 1.36 Å, Figure 4(B)). Additionally, it can be observed that EGCG and ECG shared an overlapping spatial conformation.

**Figure 5.**
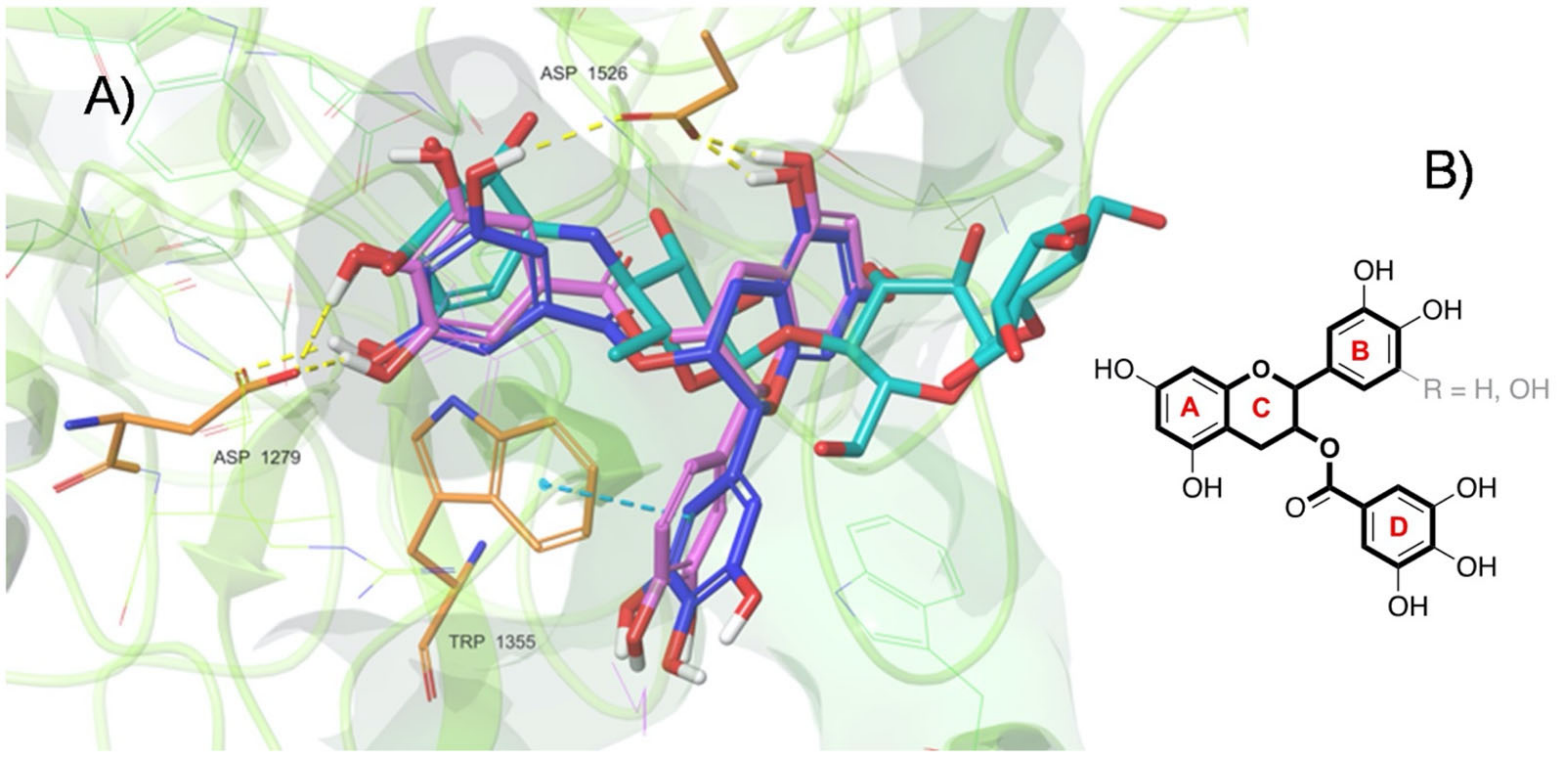
(A) Top-ranked energy pose of EGCG and ECG compounds within the acarbose-binding site of αG-C derived by in silico docking. The overall subunit is represented as a cartoon; the binding site is represented as molecular surfaces (light green); EGCG (blue), ECG (violet), co-crystallized acarbose (cyan) are in sticks; key residues are shown as orange tiny sticks. H-bonds are in yellow and pi-pi interactions are in cyan. Note EGCG and ECG T-shape and the portion of acarbose outside the binding pocket. B) Common epigallocatechin gallate skeleton indicating the conventional A-D ring system.

We found that four of the eight hydroxyl groups of EGCG and three of the seven hydroxyl groups of ECG established strong H-bonds with the sidechain function of Asp, 1279 and 1526 (the same ones involved with acarbose). Moreover, ring D of EGCG and ECG occupies the space of the unsaturated cyclitol unit of acarbose, whereas rings A and C mimic the internal rings of acarbose. Also, ECG appears well positioned inside the catalytic site because of the great stabilising effect of the arene-arene interaction found between the ring B of ECG and Trp 1355. Interestingly, a difference between EGCG and ECG, and acarbose, is due to their different overall molecular shape. In fact, while the alignment of acarbose protrudes out of the pocket, the T-shape conformation established by EGCG and ECG fully fits inside the catalytic site (Figure 5). In terms of the remaining catechins, the lack of ring D and the lower number of hydroxyl groups dramatically reduce the interactions with the enzyme. Herein, both EGC and EC showed binding modes confident with higher binding energies than ECG and EGCG, although lower than acarbose. Overall, as observed for acarbose, all catechins form H-bond with residues of Asp, in particular, 1279 and 1526 (except for EC).

**Figure 6.**
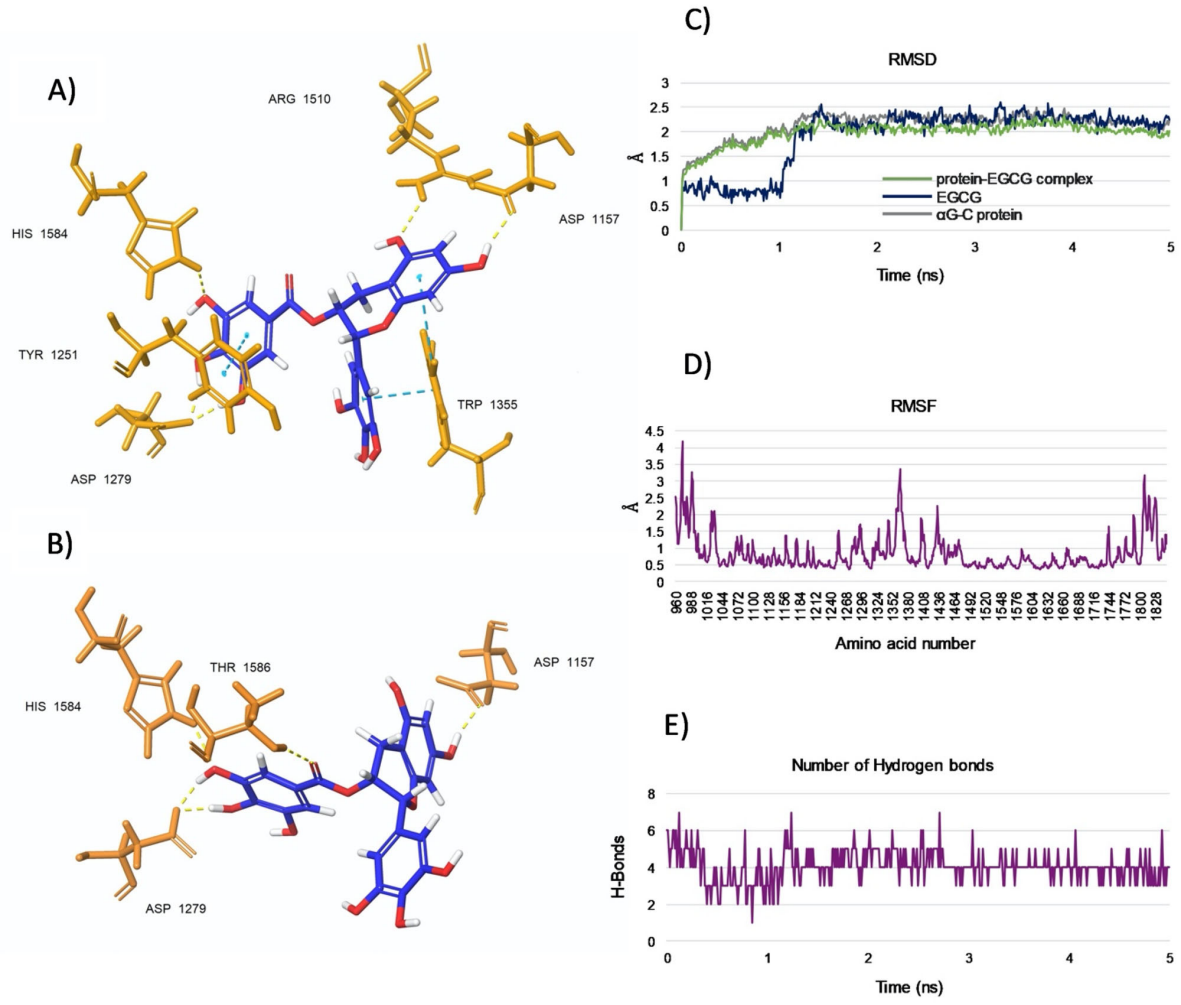
(A and B) Representative poses from two top-ranked clusters 1 and 2, respectively, displaying binding modes for EGCG within the acarbose-binding site of αG-C after MD simulation for 5 ns. The residues interacting with EGCG are shown. H-bonds are in yellow and pi-pi interactions are in cyan. (C) RMSD values from the starting protein structure (grey curve), the EGCG (blue curve) and the αG-C protein-EGCG complex (green curve) by MD simulations. Using the generated MD trajectories, RMSF (D) and HBs interactions (E) were also computed.

To predict the dynamic evolution of our system over time, we analysed the binding mode between EGCG, chosen as a model of catechin, and the target enzyme α-glucosidase by molecular dynamics (MD) simulations. According to the docking results, MD analysis revealed that EGCG aligned in the pocket retaining the observed T-shape conformation, as confirmed by representative pose for the cluster 1 (Figure 6(A)), whereas only slight differences were observed for conformers in the cluster 2 (Figure 6(B)). Specifically, for both clusters, the hydroxyl groups of the D-ring of EGCG confirmed strong H-bonds with Asp 1279 (Figures 6(A and B)), while the hydroxyls located in the A-ring did not form H-bonds with Asp 1526 but instead with the topologically adjacent residue Asp 1157. However, the pharmacophoric group on EGCG maintained the key interaction responsible for the inhibitory activity (Figures 6(A and B)). The RMSD plots disclosed that a stable complex was formed between the EGCG and αG-C protein after the simulation time period of 5 ns calculations (Figure 6C). In particular, Figure 6(C) shared that both native αG-C protein and αG-C protein-EGCG complex were stabilised after ∼1.2 ns, and retained a trend around 0.5 Å for the rest of the trajectory. This behaviour was observed also for the ligand EGCG which, after the first 1.2 ns, provided a minimal fluctuation over the rest of time. Again, a root mean square fluctuation (RMSF) value <2 Å (with the exception of residues 980–988, 1352–1380, 1800–1828) (Figure 6(D)), together to a decrease in the number of hydrogen bonds (HBs) (Figure 6E), further indicated a relative stability of the ligand-protein conformation.

## 3. Conclusions

Our results showed that the higher content of EGCG and ECG catechins in the infusion of white tea well correlated with a strong inhibition of α-glucosidase, and such inhibition was demonstrated to be more effective than acarbose. Computational studies helped us to clarify the molecular requirements for enzyme inhibition, especially for the most active catechins EGCG and ECG, as well as their specific dispositions within the active site. In particular, MD simulations confirmed the predicted binding and highlighted the relative stability of the complex formed between the model EGCG and αG-C protein.

In conclusion, our data would support the potential use of tea infusions, in particular the white tea extract, as functional food additives in dietary therapy or as an attractive strategy for managing postprandial hyperglycaemia and preventing diabetes.

## 4. Materials and Methods

### 4.1. Materials

White tea (Pai Mu Tan), Green tea (Gundpowder), and oolong tea leaves (from China) were kindly provided from Erboristeria Ghinato (Sassari, Italy). Folin-Ciocalteu’s reagent, gallic acid, α-glucosidase from Saccharomyces cerevisiae (type I; 10 U/mg protein), and p-nitrophenyl α-D-glucopyranoside (pNPG) were purchased from Sigma-Aldrich (Steinheim, Germany). Acarbose was obtained from Carbosynth Limited (Berkshire, UK). *(-)-epigallocatechin gallate* (EGCG) (98%), *(-)-epigallocatechin* (EGC) (98%), *(-)-epicatechin gallate* (ECG) (98%), and *epicatechin* (EC) (98%) were supplied by Zhejiang Yixin Pharmaceutical Co., Ltd. (Lanxi, Zhejiang, China). All other chemicals were analytical grade and were used without further purification.

### 4.2. Preparation of tea infusions

Green, white, and oolong tea leaves were milled into a fine powder by using a Sterilmixer lab (VWR International, Italy). Tea infusions were prepared by a traditional method of pouring 100 ml of water at 100 °C on 5.0 g of tea powder placed into a tea infuser and brewed for 5 min. A portion of samples was then filtered through Whatman No.1 and stored at 4 °C until use. Another portion of the extract was freeze-dried and weighed to determine the concentration recovered from extracts (g/L). The lyophilised infusions were kept in a desiccator and protected from light until analysis.

### 4.3. Analysis of total phenolic content (TPC)

The TPC of the tea infusions was determined using the Folin-Ciocalteu method as previously described^42^^,^^43^. 100 μL of each tea extract, previously diluted 20-fold in distilled water, were added to 0.5 ml of Folin-Ciocalteu reagent 10-fold diluted. After 1 min of mixing, 1 ml of sodium carbonate solution (7.5%, w/v) was added. The mixture was left 30 min at 30 °C in the dark and absorbance at 760 nm was measured. The calibration curve of absorbance vs concentration of gallic acid (GA) used as standard in the range 25–500 mg/L (0.0058*x* + 0.0355; *R*^2^ = 0.9994) was used for quantification of TPC, and results were expressed as milligrams of gallic acid equivalent (GAE) per gram of extract.

### 4.4. HPLC analysis of catechins content

Catechins extracted from tea leaves were identified and quantified using a modified HPLC method, as previously described^29^^,^^31^. Stock solutions of EGCG, ECG, EGC, and EC standards were individually prepared by dissolving appropriate amount of reference substance in water and then diluted serially in water to yield serial mixture standard solutions containing appropriate concentrations of the above catechins (1.0, 2.5, 5.0, 10.0, and 50.0 μg/mL of EGCG; 0.5, 1.25, 2.5, 5.0, and 25.0, μg/mL of ECG, EGC and EC). All the standard solutions were stored at −20 °C until analysis. A 20 μL aliquot of standard solutions and sample extract was injected and analysed two times using HPLC, and the standard curves were plotted as peak areas versus concentration. HPLC was performed using a Flexar HPLC system (PerkinElmer Inc., Waltham, MA) by using a reversed-phase column (250 mm × 4.6 mm i.d., 5 μm) Restek ultra C18 (Restek Corporation, Bellefonte, PA) at 280 nm, an elution flow rate of 1.5 ml/min, and a linear solvent gradient of A-B (A, 10 mM KH2PO4 (pH 4.0); B, CH3CN/H2O (65%/35%) as follows: 0 min, 20% B; 5 min, 20% B; 15 min, 30% B; 40 min, 60% B). TotalChrome software (PerkinElmer) was used for data acquisition and data processing. The individual catechins were quantified in extracts by comparison with the generated standard curves. The catechin content of the teas was expressed as a percentage by mass on a sample dry matter basis.

### 4.5. Alpha-Glucosidase inhibition assay

The effect of the tea extracts (GTI, WTI, OTI) on α-glucosidase activity was determined according to the method previously described with minor changes^38^. The substrate solution p-nitrophenyl glucopyranoside (pNPG) (3.0 mM) was prepared in 20 mM phosphate buffer, pH 6.9. One hundred μL of α-glucosidase (0.1 U/mL) were mixed with 50 μL of the different concentrations of the extracts (0.25–0.016 mg/mL) or catechins (EGCG and ECG 0.25–0.016 mg/mL, and EGC 1.0–0.20 mg/mL) and the mixture was incubated at 37 °C for 10 min. After pre-incubation, 50 μL of pNPG solution was added to start the reaction. The reaction mixture was incubated at 37 °C for an additional 10 min and stopped by adding 2 ml of 0.1 M Na_2_CO_3_. The α-glucosidase activity was determined by measuring the yellow-colored para-nitrophenol released from pNPG at 405 nm. The α-glucosidase inhibition activity was calculated as follows:

% Inhibition=[(Ac – As)/Ac]×100

where Ac and As are the absorbance of the control and the tested samples, respectively. Commercial inhibitor acarbose (20–1.0 mg/m) was used as a positive control and distilled water as a negative control. All samples were assayed in triplicate.

As a measure of the potency of the inhibitors tested, IC_50_ values were calculated from the enzyme activity data. These are defined as the concentration of an inhibitor required for reducing 50% of the enzyme activity obtained from an activity vs concentration plot. The data points were fitted into a nonlinear sigmoid plot to consider the non-linear concentration dependence of enzyme inhibitor interaction at low and high concentrations^44^.

### 4.6. Computational methods

Molecular docking calculations were performed on tea catechins in order to evaluate their interactions in the α-glucosidase active site. AV and AD4 were used as docking software. X-ray crystallographic structure of the C-terminal subunit of human α-glucosidase in complex with acarbose was retrieved from PDB Data Bank (PDB code: 3TOP)^45^.

For protein preparation, USFC Chimaera v1.16^46^ was used to remove water, the chains A, B and D from the pdb file. ADT module of MGLTools v1.5.7 was used to add polar hydrogens and Kollman charges to the protein. ADT was also used to create a grid box (coordinate: x=-31,528, y = 35,624, z = 26,388; size x = 40; y = 40; z = 40; default grid spacing of 0.375 Å), based on the position of co-crystallized ligand acarbose. We next used Pybel^47^ Python script to prepare the ligands from the database containing tea catechins SMILES. The 3D structure for each was obtained, which was then submitted to energetic minimisation (force field MMFF94s, 10000 steps) and saved in pdb format file, while ADT was used to assign Gasteiger charges and convert the ligands in the pdbqt format. Rigid receptor flexible ligand docking was performed firstly with AV and next with AD4 using Lamarckian Genetic Algorithm (number of GA = 50; population size = 300). The common ligand-interactions in each complex were scrutinised using Free Maestro^48^.

Molecular dynamics (MD) simulation was performed on the selected best-docked pose of EGCG in the catalytic domain of α-glucosidase active site (C-terminal subunit of human α-glucosidase). This analysis was carried out using GROMACS v2022.3^49^^,^^50^ as an MD engine, using Google Colab as high-performance computing tool for calculation. The Charmm36^51^ force field was selected and the ligand topologies were generated from CgenFF. The ligand-protein complex was solvated using water model TIP3P in a 10Å cubic box edge. The simulation system was set up electrically neutral for calculation. The solvated system was subjected to energy minimisation for 5000 steps. The statistical ensemble such as NVT (constant number of particles, volume, and temperature), NPT (constant number of particles, pressure, and temperature), and the production run were conducted for 100 ps in the MD simulation. Then, MD simulation was carried out at 300 K for the time duration of 5 ns (nanoseconds). Finally, root mean square deviation (RMSD) to determine the stability of the complex, and root mean square fluctuation (RMSF), as well as hydrogen bond parameters were performed to further evaluate ligand-protein stability.

### 4.7. Statistical analysis

All data were expressed as the mean ± standard deviation of three replications of the experiment. Significance was assessed by one-way analysis of variance (ANOVA) (GraphPad Prism 5, San Diego, CA, USA). Individual differences were evaluated using a nonparametric *post hoc* test (Tukey’s test) and considered statistically significant at *p* < 0.05.
